# Expression of interferon-*γ* in human adrenal gland and kidney tumours

**DOI:** 10.1038/sj.bjc.6603870

**Published:** 2007-07-10

**Authors:** Q Li, X-q Zhang, L Nie, G-s Chen, H Li, F Zhang, L-y Zhang, L Hong, S-f Wang, H Wang

**Affiliations:** 1 State Key Laboratory of Cancer Biology and Department of Pathology, Xijing Hospital, Fourth Military Medical University Xi'an 710032, China; 2 Department of Medical Biochemistry and Microbiology, Box 582, Biomedical Center, Uppsala S-751 23, Sweden; 3 Department of Urinary Surgery, Xijing Hospital, Fourth Military Medical University Xi'an 710032, China

**Keywords:** interferon-*γ*, adrenal tumour, clear cell renal cell carcinoma

## Abstract

It is known that interferon-*γ* (IFN-*γ*) is produced by activated T and NK lymphoid cells, mononuclear cells, and macrophage and dendritic cells. Our previous studies have shown that IFN-*γ*-like immunoreactivity also appears in human adrenal cortical tumour and phaeochromocytoma. To investigate whether human tumour cells can produce IFN-*γ*, we examined 429 biopsy specimens of 30 kinds of tumour and tumour-surrounding tissues in adrenal glands and in kidneys by using immunohistochemistry and *in situ* hybridisation. IFN-*γ* immunoactivity was shown in 34.3% of the adrenal cortical adenomas, 50% of the adrenal cortical carcinomas, 26.7% of the phaeochromocytomas, 26.7% of the clear cell renal cell carcinomas (RCCs), 22% of the adrenal cortexes and 40% of medullas adjacent to tumours. The positive samples and expression areas were well overlapped between the IFN-*γ* mRNA and the immunohistochemistry staining. Western blot analysis has further confirmed the immunohistochemistry results by showing a distinct IFN-*γ* band corresponding to 17.4 kDa in tissue extracts from adrenal cortical adenoma, phaeochromocytoma and clear cell RCCs. These results indicate that IFN-*γ* is produced by some types of tumour cells, suggesting it may play a dual role in the development of these tumours.

Interferon-*γ* (IFN-*γ*) is a multi-functional cytokine produced mainly by macrophages, dendritic cells, activated T lymphocytes and NK cells (Tripp *et al*, 1993; Gessani and Belardelli, 1998; Fukao *et al*, 2000; Murphy *et al*, 2000). In addition to its well-known antiviral activities, IFN-*γ* also plays a role in host antitumor responses. Endogenously produced IFN-*γ* is reported to function in cancer immunosurveillance, protecting the host against the growth of transplanted tumours and the formation of primary, chemically induced spontaneous tumours (Shankaran *et al*, 2001; Street *et al*, 2001). Mice deficiency of IFN-*γ* signalling developing spontaneous tumours at a higher frequency than normal mice further indicates that IFN-*γ* is involved in immune response to tumours (Kaplan *et al*, 1998). IFN-*γ* also regulates cell proliferation and apoptosis via activating the downstream JAK–STAT signalling pathway (Buard *et al*, 1998; Haque and Williams, 1998; Weber-Nordt *et al*, 1998; Lee *et al*, 2000). IFN-*γ*-like immunoreactivity has been proved to appear in human adrenal cortical tumours, phaeochromocytomas and hepatocellular carcinomas (HCCs) by immunohistochemical method (Li and Song, 1995, 1996; Chia *et al*, 2002). However, immunohistochemistry analysis alone cannot confirm whether tumour cells produce IFN-*γ*. To answer this question, we used immunohistochemistry combined with *in situ* hybridisation and Western blot method to screen a cluster of different types of tumours.

## MATERIALS AND METHODS

### Biopsy material

A total of 429 biopsy specimens of 30 kinds of tumour and three types of tumour-surrounding tissues were collected from patients who had not received any chemotherapeutic or immunomodulatory treatment before operations. Tumour tissues were classified according to the classification system of the World Health Organization (Table 1). Tissue specimens were fixed for 18–24 h in 10% buffered formalin and routinely processed to paraffin embedding.

### Immunohistochemistry

Serial sections from each case were stained with a mouse monoclonal antibody specific to human IFN-*γ* (United States Patent 4599306, Department of Immunology, Fourth Military Medical University, Xi'an, China). This antibody has optimised specificity by using the method of microwave antigen retrieval on formalin-fixed, paraffin-embedded tissue. Immunohistochemical staining was performed using a standard streptavidin–biotin–peroxidase complex (SABC) (BOSTER Incorporation, Wuhan, China). Visualisation was performed using diaminobenzidine (DAB) for the horseradish peroxidase. The sections were counterstained with haematoxylin, dehydrated and mounted. A positive control was performed using the L26 (anti-CD20) monoclonal antibody (Dako, Glostrup, Denmark) a marker of B lymphocytes. Negative controls were obtained by replacing the primary antibody with non-immune mouse serum.

### Immunoblotting

Fresh tissue samples from six cases of adrenal cortical adenomas, five cases of phaeochromocytomas, five cases of clear cell renal cell carcinomas (RCCs) and one case of colonic adenocarcinoma were cut into small pieces and homogenised using a Polytrope high-speed metal homogeniser (Brinkmann Instruments, Westbury, NY, USA) in five volumes of 50 mmol l^−1^ Tris buffer (pH 7.7) containing 0.5 mmol l^−1^ phenylmethylsulphonylfluoride to inhibit proteolysis. The supernatant was obtained after centrifugation at 12 000 r.p.m. min^−1^ for 15 min at 4°C. A volume of 1 *μ*l supernatant from each sample was dot blotted on a polyvinylidene fluoride (PVDF) paper. Immunostaining was performed using a standard SABC (BOSTER Incorporation). Negative control was obtained by replacing the primary antibody with non-immune mouse serum.

### Sodium dodecyl sulfate–polyacrylamide gel electrophoresis and Western blot analysis

The dot blotting positive samples (two from adenomas, one from phaeochromocytoma and one from clear cell RCCs) and one negative colonic carcinoma were separated by 15% SDS–polyacrylamide gels (PAGE). Molecular weight markers (C-6210; Sigma Chemical Co, St Louis, MO, USA) were run together with the samples. After the separation, the proteins were electroblotted onto PVDF paper utilising the Phast system. The PVDF membranes were blocked by incubation in 0.02 mol l^−1^ Na_2_HPO_4_, 0.15 mol l^−1^ NaCl, pH 7.2 containing 0.05% Tween 20 and 3% bovine serum albumin for 1 h at room temperature and then followed by immunoblotting. Negative control was obtained by replacing the primary antibody with non-immune mouse serum.

### *In situ* hybridisation analysis

*In situ* hybridisation for IFN-*γ* mRNA was performed on further serial sections from 35 cases of adrenal cortical adenomas, four cases of adrenal cortical carcinomas, 30 cases of phaeochromocytomas, 60 cases of clear cell RCCs, 10 cases of HCCs, nine cases of tumour surrounding adrenal cortexes, five cases of medullas and 23 cases of tumour around renal tissues by using an oligonucleartide IFN-*γ* kit (BOSTER Incorporation). The experiment was performed according to the instruction. Briefly, after de-waxing and re-hydration, sections were pretreated with 0.3% hydrogen peroxide to suppress the endogenous peroxide. Tissues were postfixed with 1% paraformaldehyde and were further reacted with prehybridisation solution. A volume of 30 *μ*l hybridisation solution was supplied on each tissue section with glass coverslips and incubated overnight at 38–42°C. Coverslips were removed and the sections were washed. After blocking solutions, the sections were incubated with biotinilated mouse-anti-digoxingin for 1 h at 37°C. The sections were then washed thoroughly and incubated with SABC at room temperature. Visualisation was performed by using DAB. The sections were counterstained with haematoxylin, then dehydrated and mounted.

Controls were processed with the omission of the probe and with mRNase before hybridisation.

### Scoring of immunohistochemistry and *in situ* hybridisation

The immunoreactivity and *in situ* hybridisation were scored according to the staining extension and intensity. The intensity of staining was scored from 0 to 3: 0 for negative; 1 for weak; 2 for moderate; 3 for strong staining. The extension of immunoreactivity was estimated from 0 to 4: 0 for negative; 1 for 1–25%; 2 for 26–50%; 3 for 51–75%; 4 for 76–100% of positive immunoreactivity cells. The positive immunoreactivity or *in situ* hybridisation staining was determined by the combined staining score (intensity score plus extent score): 0 for ⩽2; 1 for 3; 2 for 4–5; 3 for 6–7.

## RESULTS

### Immunohistochemistry

The immunoreactive IFN-*γ*-positive cells displayed distinct brown staining; immunoreactive materials appearing as densely packed granules in the entire or focal of cytoplasm, whereas immunostaining was undetectable in negative control sections, indicating that IFN-*γ* immunoreactive staining was specific.

**Figure 1:**
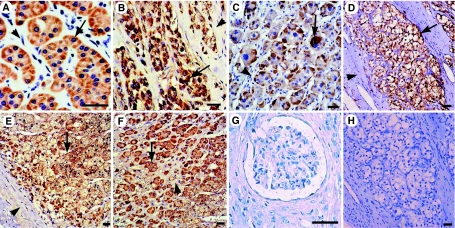
IFN-*γ* immunoreactivity in (**A**) adrenal cortical adenoma, (**B**) cortical carcinoma, (**C**) phaeochromocytoma, (**D**) clear cell RCC, tumour adjacent tissue in (**E**) adrenal cortex (**F**) and medulla. The immunoreactive IFN-*γ*-positive cells show distinct brown staining. The immunoreactive materials often appear as densely packed granules filling the entire or focal of cytoplasm of tumour cells (arrow) and the interstitial tissue is negative (arrowhead). No IFN-*γ* immunoreactivity is seen in (**G**) kidney. Clear cell RCC (same case as in **D**) matched (**H**) negative control staining do not show any positive staining. Bar=50 *μ*m.

**Figure 2:**
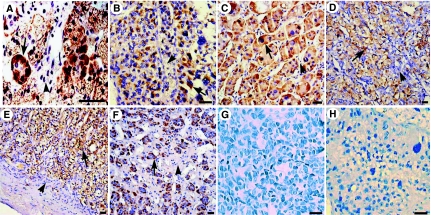
*In situ* hybridisation for IFN-*γ* mRNA. The IFN-*γ* mRNA signals are seen in adrenal (**A**) cortical adenoma, (**B**) cortical carcinoma, (**C**) phaeochromocytoma, (**D**) clear cell RCC, tumour adjacent tissue (**E**) in adrenal cortex (**F**) and medulla. IFN-*γ* mRNA is apparent in the cytoplasm of parenchyma cells (arrow), not in interstitial tissue (arrowhead). IFN-*γ* mRNA signals are not seen in HCC (**G**). Adrenal cortical carcinoma (same case as in **B**) matched negative control with (**H**) mRNase do not show any positive staining. Bar=50 *μ*m.

Prominent staining cells for immunoreactive IFN-*γ* was observed in 12 out of 35 (34.3%) of the adrenal cortical adenomas, two of four (50%) of the adrenal cortical carcinomas, eight out of 30 (26.7%) of the phaeochromocytomas and 16 out of 60 (26.7%) of the clear cell RCC. The IFN-*γ* immunoreactivity was mainly restricted to tumour cells, and was also in some lymph cells and macrophages but was usually not in other non-tumour cells. The IFN-*γ* immunoreactivity was also demonstrated in two out of nine (22%) of the adrenal cortex and two out of five (40%) of the medulla tissues adjacent to tumours (Figures 1 and 2 and Table 2). However, IFN-*γ* immunoreactivity could not be detected in renal tissues surrounding clear cell RCCs. Surprisingly, the rest 260 tumour biopsies, originating from other organs than adrenal gland and kidney, did not show IFN-*γ* immunostaining in any single case.

### Western blot analysis

**Figure 3:**
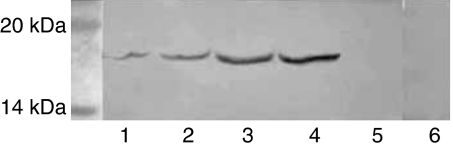
Western blot analysis for IFN-*γ*. A 17.4 kDa IFN-*γ* protein band appears in the extracts from adrenal cortical adenomas (lanes 1 and 2), clear cell RCC (lane 3) and pheochromocytoma (lane 4). Negative control (lane 5, same case as in lane 1) and colonic adenocarcinoma (lane 6) do not show any band.

A distinct IFN-*γ* band corresponding to 17.4 kDa (Figure 3) was observed in tissue extracts from two out of six cases of the adrenal cortical adenomas, one out of five cases of the phaeochromocytomas and one out of five cases of the clear cell RCCs. Colonic adenocarcinoma and negative control did not show positive staining.

### *In situ* hybridisation analysis

IFN-*γ* mRNA signals could only be seen in the sections reacted with antisense probe. It has no staining in control sections, which either have omitted IFN-*γ* probe or have prior incubated with mRNase. Prominent cytoplasmic staining for IFN-*γ* mRNA were detected in 13 out of 35 of the adrenal cortical adenomas, two out of four of the cortical carcinomas, nine out of 30 of the phaeochromocytomas, 16 out of 60 of the clear cell RCC, two out of nine of the tumour adjacent tissues in adrenal cortexes and two out of five of the medullas. No IFN-*γ* mRNA was detected in the renal tissues surrounding clear cell RCC. The IFN-*γ* expressing samples and areas were well overlapped in between of mRNA signal and immunohistochemistry staining (Figure 2).

## DISCUSSION

The adrenal gland consists of two distinct types of primary tumours: one arising from the adrenal cortex, including adenomas and carcinomas, and the other from the medulla representing neural crest-derived chromaffin cell tumours (pheaochromocytomas). RCC is a group of malignancies arising from the epithelium of the renal tubules. Clear cell RCC, one type of RCC, is composed of cells with clear or eosinophilic cytoplasm within a delicate vascular network.

Our previous study has demonstrated that IFN-*γ*-like immunoreactivity appeared in the cytoplasm of tumour cells in adrenal cortical adenoma, cortical carcinoma and pheochoromocytoma by using DB1 monoclonal antibody (Li and Song, 1995, 1996). The IFN-*γ*-like immunoreactivity is not a non-specific binding, since no staining was detected in the absence of primary antibody. In addition, DB1 monoclonal antibody is a high-affinity antibody against rat IFN-*γ* with specific labelling of different molecular forms of recombinant rat IFN-*γ*, inhibiting biological effects of IFN-*γ* *in vitro* and *in vivo* (Jacob *et al*, 1987; van der Meide *et al*, 1986). However, DB1 is a monoclonal antibody against rat IFN-*γ* but not human IFN-*γ*. In the present study, a monoclonal mouse-anti-human IFN-*γ* antibody was used to characterise the IFN-*γ* immunoactivity in 429 human biopsy samples from 30 different types of tumours and tumour-surrounding tissues in adrenal glands and in kidneys. The results showed that 34.3% of the adrenal cortical adenomas, 50% of the adrenal cortical carcinomas, 26.7% of the phaeochromocytomas and 26.7% of the clear cell RCC expressed IFN-*γ* immunoactivity. IFN-*γ* was not detectable in other types of tumour. Western blot analysis revealed IFN-*γ* band corresponding to 17.4 kDa in tissue extracts from two out of six cases of the adrenal cortical adenomas, one out of five cases of the phaeochromocytomas and one out of five cases of the clear cell RCCs, whereas the colonic adenocarcinoma and the negative control did not show any band. The results suggest that the IFN-*γ* immunoactivity is specific.

To confirm that the IFN-*γ* is indeed produced in tumour cells, IFN-*γ* mRNA *in situ* hybridisation was performed on the same biopsy samples. The *in situ* hybridisation results confirmed our hypothesis that the transcripts of IFN-*γ* genes were inside the adrenal and RCC tumour cells. The mRNA expressing tissues were overlapped with the immunohistochemistry staining, indicating that the IFN-*γ* protein was generated by tumour cells. IFN-*γ* protein and mRNA levels were generally higher in adrenal cortical adenomas than in cortical carcinomas. Clear cell RCCs expressed both mRNA and IFN-*γ* protein, but not in the tumour-surrounding renal tissue. Therefore, IFN-*γ* expression may in some way correlated to the tumour malignancy level or cell differentiation.

It remains unclear why IFN-*γ* was expressed in 22% of tumour surrounding tissue in adrenal cortexes and 40% of in medullas. Cells with IFN-*γ*-like immunoreactivity have also been reported in type II alveolar epithelial cells in interstitial lung disease (Wallace and Howie, 1999), rat neurons (Kiefer *et al*, 1991), rat skeletal muscle (Nennesmo *et al*, 1989), transplanted rat heart tissue (Wanders *et al*, 1992), astrocytes in active chronic multiple sclerosis lesion (Traugott and Lebon, 1988) and cerebrovascular endothelial cells from aged mice (Wei *et al*, 2000). However, there was no further report about the significance of the IFN-*γ* expression in those tissues or cells. IFN-*γ* has also been reported weakly expressed in HCCs (Chia *et al*, 2002). But in the present study, neither protein nor mRNA of IFN-*γ* was expressed in the 10 cases of HCCs, probably because that the methods we used are not sensitive enough.

It is well known that IFN-*γ* contributes to the increased antitumour activity of immune cells, though the reason why some tumour cells produce IFN-*γ* is not clear. Previous studies have established that testicular germ cell tumour cells can express both mRNA and protein of IFN-*γ* and the expressed IFN-*γ* is able to induce the chemokine IP-10, indicating its biological activity, whereas it is not able to phosphorylate the downstream STAT1 (Schweyer *et al*, 2002, 2003). A number of tumours and tumour cell lines have also been reported to develop a permanent and selective IFN-*γ* insensitivity, such as testicular germ cell tumours, RCC cell line, basal cell carcinoma of the skin and human lung adenocarcinoma cell lines (Kooy *et al*, 1998; Dovhey *et al*, 2000; Nagao *et al*, 2000; Schweyer *et al*, 2002, 2003; Dunn *et al*, 2005b). Such insensitivity to IFN-*γ* is due to lack of expression of IFN-*γ* receptors or the receptor downstream genes. Tumour cells can naturally develop mutations on IFN-*γ* downstream target genes, such as JAK and Stat1, to render them insensitive to IFN-*γ*.

Recently, a concept of cancer immunoediting has been put forward (Ikeda *et al*, 2002; Dunn *et al*, 2004a, 2004b, 2005a, 2005b, 2006). It is a process consisting of three phases: elimination (i.e., cancer immunosurveillance), equilibrium and escape. The immune system not only protects the host against development of primary nonviral cancers but also sculpts tumour immunogenicity. Thus, IFN-*γ* may play a dual role in preventing development of primary and transplanted tumours on one hand, and sculpting the immunogenic phenotype of tumours on the other. Tumour escape may be attributable to tumour genetic changes that affect tumour recognition by immune effective cells (such as, loss of antigen expression, loss of MHC components, shedding of NKG2D ligands and development of IFN-*γ* insensitivity) or provide tumours with mechanisms to escape immune destruction (such as, defects in death-receptor signalling pathways or expression of antiapoptotic signals including constitutively active STAT3). The dysregulation of MHC class I processing and presentation, or/and development of IFN-*γ* insensitivity in tumour cells, would allow tumours to escape from the elimination phase of the cancer immunoediting process (Dunn *et al*, 2004b). However, whether the specific expression of IFN-*γ* in tumours from adrenal gland and kidney is correlated with tumour escape is not clear and needs to be further elucidated.

In conclusion, we provide solid evidence that tumour cells express IFN-*γ* protein and mRNA especially in adrenal gland and kidney tumours. Our findings give a further insight into the role of IFN-*γ* in the pathogenesis of tumour, which will be critical for effective immunotherapeutic approaches for cancer therapy in the future.

## Figures and Tables

**Table 1:** Biopsy specimens

				**Age**	
**Histopathological diagnosis**	**Total case number**	**Male**	**Female**	**Median**	**Range**
Tumour around adrenal cortex	9	6	3	43	28–56
Tumour around adrenal medulla	5	4	1	41	26–48
Adrenal cortical adenoma	35	21	14	45	28–64
Adrenal cortical carcinoma	4	4	0	49	35–57
Phaeochromocytoma	30	17	13	41	12–48
Tumour around kidney	23	19	4	54	51–68
Clear cell RCC	60	47	13	56	42–73
Hemangioma	10	4	6	21	12–36
Fibroadenoma of breast	10	0	10	49	35–59
Ovarian teratoma	10	0	10	29	19–36
Leiomyoma	10	0	10	43	26–56
Pituitary adenoma	10	0	10	38	32–47
Thyroid adenoma	10	2	8	36	21–43
Intestinal adenoma	10	7	3	29	16–49
Ovarian adenoma	10	0	10	23	18–31
Cutaneous papilloma	10	6	4	32	16–46
Gastric carcinoma	10	6	4	52	38–73
Intestinal carcinoma	10	7	3	47	29–69
Breast carcinoma	10	8	2	56	39–76
Thyroid carcinoma	10	3	7	43	34–58
Pancreatic carcinoma	10	7	3	56	43–74
Bladder carcinoma	10	10	0	63	41–78
Larynx squamous carcinoma	10	10	0	51	35–67
Squamous carcinoma of skin	10	4	6	47	44–63
Cervical squamous carcinoma	10	0	10	54	41–69
Nasopharyngeal carcinoma	10	9	1	46	32–58
Lung cancer	10	6	4	62	24–84
Meningeoma	10	6	4	32	14–37
Astrocytoma	10	7	3	48	16–70
Rhabdomyosarcoma	10	7	3	12	6–48
Osteosarcoma	10	10	0	19	14–33
Fibrosarcoma	10	5	5	16	17–42
Hepatocellular carcinoma	10	4	6	43	34–51

RCC, renal cell carcinomas.

**Table 2:** IFN-*γ* immunoreactivity in tumours and tumours adjacent tissues

		**Score**					
**Type**	**Case no.**	**0**	**1**	**2**	**3**	**Positive no.**	**%**
ACA	35	23	2	7	3	12	34.3
ACC	4	2	0	0	2	2	50.0
Phaeo	30	22	0	4	4	8	26.7
CRCC	60	44	5	8	3	16	26.7
TAC	9	7	0	0	2	2	22.2
TAM	5	3	0	1	1	2	40.0

AC, tumour around adrenal cortex; ACA, adrenal cortical adenoma; ACC, adrenal cortical carcinoma; Phaeo, phaeochromocytoma; CRCC, clear cell renal cell carcinoma; IFN-*γ*, interferon-*γ*; TAM, tumour around adrenal medulla.
